# Revealing changes in molecular composition of plant cell walls on the micron-level by Raman mapping and vertex component analysis (VCA)

**DOI:** 10.3389/fpls.2014.00306

**Published:** 2014-06-30

**Authors:** Notburga Gierlinger

**Affiliations:** ^1^Department of Materials Science and Process Engineering, BOKU-University of Natural Resources and Life ScienceVienna, Austria; ^2^Institute for Building Materials, Eidgenössische Technische Hochschule ZurichZurich, Switzerland; ^3^Applied Wood Research Laboratory, Empa – Swiss Federal Laboratories for Material Testing and ResearchDuebendorf, Switzerland

**Keywords:** secondary cell wall, wood, lignin, cellulose, beech, spruce, Raman microscopy, VCA

## Abstract

At the molecular level the plant cell walls consist of a few nanometer thick semi-crystalline cellulose fibrils embedded in amorphous matrix polymers such as pectins, hemicelluloses, and lignins. The arrangement of these molecules within the cell wall in different plant tissues, cells and cell wall layers is of crucial importance for a better understanding and thus optimized utilization of plant biomass. During the last years Confocal Raman microscopy evolved as a powerful method in plant science by revealing the different molecules in context with the microstructure. In this study two-dimensional spectral maps have been acquired of micro-cross-sections of spruce (softwood) and beech (hardwood). Raman images have been derived by using univariate (band integration, height ratios) and multivariate methods [vertex component analysis (VCA)]. While univariate analysis only visualizes changes in selected band heights or areas, VCA separates anatomical regions and cell wall layers with the most different molecular structures. Beside visualization of the distinguished regions and features the underlying molecular structure can be derived based on the endmember spectra. VCA revealed that the lumen sided S3 layer has a similar molecular composition as the pit membrane, both revealing a clear change in lignin composition compared to all other cell wall regions. Within the S2 layer a lamellar structure was visualized, which was elucidated to derive from slight changes in lignin composition and content and might be due to successive but not uniform lignification during growth.

## INTRODUCTION

Plant cell walls comprise very complex and highly variable structures. Their basic structural elements are crystalline cellulose [(1-4)-β-linked glucose] microfibrils embedded in matrix polymers. In the thin primary cell wall (0.1–1 μm) built during early growth a network is formed together with pectins, hemicelluloses, and proteins (Keegstra, 2010). These thin walls give already shape and structure to plant cells, tissues, and ultimately organs and are sufficiently strong to prevent the cell from rupturing, yet they must be flexible and plastic to accommodate growth (Cosgrove, 2005). The secondary cell wall, a thick layer which is deposited after cells cease enlargement, is often lignified. The deposition of lignin in the plant cell wall has been considered to be one of the key factors that allowed land plants to flourish in terrestrial ecosystems (Weng and Chapple, 2010). Lignins are three-dimensional, amorphous heteropolymers that result from the oxidative coupling of three *p*-hydroxycinnamyl alcohols (*p*-coumaryl, coniferyl, and sinapyl alcohols) in a reaction mediated by roughly ten enzymes leading to the formation of guaiacyl (G), syringyl (S), and hydroxyphenyl (H) subunits (Boerjan et al., 2003). The cross-coupling reaction produces an hydrophobic heteropolymer, that imparts water impermeability and confers structural support and flexural stiffness to the plant cell wall (Vanholme et al., 2010). The nature and randomness of the linkages make lignin to one of the hardest biopolymers to degrade (Ralph et al., 1998). The amount and molecular structure of the lignin in the cell walls is indeed one of the limiting factors that determine the digestibility of forage crops (Bhatt et al., 2008; Studer et al., 2011; Yoo et al., 2012). Lignins are therefore an undesirable component not only in the pulp industry, but also in the biofuel production (Ralph et al., 2004; Pu et al., 2011; Yoo et al., 2012; Kim and Ralph, 2014). Given the advances in biotechnology and genetics, it is only natural that these tools are explored to improve the desired feedstock properties, e.g., enhance the biological conversion properties of lignocellulosic crops (Lu et al., 2009). Downregulating the enzymes of the major lignin pathways can affect the course and/or extent of lignification and gives expanded opportunities for engineering the composition and consequent properties of lignin for improved utilization of valuable plant resources (Boerjan et al., 2003; Artz et al., 2008; Gonzalez-Guerrero et al., 2008; Vanholme et al., 2010; Li et al., 2011; Delgado-Cerezo et al., 2012; Thomas et al., 2013; Kim and Ralph, 2014).

Lignified cell walls represent the major proportion of the terrestrial plant biomass and an increasing demand for natural as well as “designer” plant material is observed (Boudet et al., 2003; Pu et al., 2011). For an optimized production and utilization knowledge on the natural and modified material is of extreme importance. Due to the complex nature of plant cell walls knowledge on chemical composition as well as the structural arrangement at the different hierarchical levels (tissue, cell-micro and nano-level) is of importance as all together influence the properties. To reveal chemical information fast within the native cell wall different spectroscopy approaches have been developed (e.g., McCann et al., 1992; Oliveira et al., 2009; Robinson and Mansfield, 2009; Krongtaew et al., 2010; Lyubenova et al., 2012; Abidi et al., 2014). In combination with microscopy especially Confocal Raman imaging evolved as an important tool as molecular vibrations are monitored in context with the cell wall structure and thus the organization of components within the native wall of single cells is revealed (Agarwal, 2006; Gierlinger and Schwanninger, 2007; Gierlinger, 2011; Sun et al., 2011a; Gierlinger et al., 2013). Important information on cellulose microfibril orientation (Gierlinger et al., 2010b) and on the matrix polymers lignin and pectin can be revealed at once, so that a comprehensive picture of cell wall design is gained (Gierlinger and Schwanninger, 2006; Hanninen et al., 2011; Richter et al., 2011; Gierlinger et al., 2012; Perera et al., 2012).

Plant cell wall spectra are known to comprise broad and overlapping bands due to the intimate mixture of the different polymers and components and if the imaging approach is applied thousands of them are acquired (Gierlinger et al., 2012). Therefore data analysis of single spectra and single wavenumbers or bands has limitations and multivariate data analysis approaches high potential in elucidating changes in molecular structure (Shinzawa et al., 2009; Perera et al., 2011). In this paper vertex component analysis (VCA) is introduced as a promising approach for analyzing Raman maps of wooden cell walls. So far VCA has been successfully applied in the analysis of biomedical Raman images (Hedegaard et al., 2011; Krafft et al., 2011, 2012; Bergner et al., 2012; Ashtikar et al., 2013; Lattermann et al., 2013; Mazur et al., 2013), but not on plant cell walls. VCA belongs to the group of multivariate curve resolution methods and projects the data to the identified orthogonal subspace in an interactive way and finds the endmember by repeated iteration (Nascimento and Dias, 2005; Zhang and Tauler, 2013). The endmember spectra have been proposed to refer to the pure constituent spectra present in the image pixel (Zhang and Tauler, 2013). With this paper the feasibility of VCA to get new insights into the molecular structure of plant cell walls will be tested on Raman maps of softwood (spruce) and hardwood (beech). As the pure polymer and component might not be present at any single pixel of the Raman map not only pure components and endmember spectra are expected. The aim is therefore to differentiate first of all cell wall regions different in molecular structure and then get new insights into composition of these defined areas based on the analysis of the endmember spectra.

## MATERIALS AND METHODS

### SAMPLE PREPARATION

From fresh spruce (*Picea abies* Karst) and beech (*Fagus sylvatica*) 8 μm thick cross sections were cut using a rotary microtome (LEICA RM2255, Germany). Placed on a glass slide a drop of D_2_O and a coverslip is added and sealed with nailpolish to avoid evaporation of D_2_O during the measurement.

### RAMAN IMAGING

The Raman imaging measurements were performed with an InVia Renishaw Confocal Raman spectroscope (Renishaw, UK) using a 532 nm laser, an oil immersion objective (Nikon, 100x, NA = 1.4) and 1800 l/mm grating. For mapping a step size of 0.2 μm (spruce) and 0, 3 μm (beech) and an integration time of 0.4 s was chosen in the StreamlineHR mode. After data acquisition a cosmic ray removal filter was applied using the Wire 3.7 software (Renishaw UK) and afterward data exported to Cytospec (v. 2.00.01) for univariate and multivariate data analysis data. Univariate data analysis was performed by calculating band areas and band height ratios of different wavenumber regions.

Different multivariate methods (principal component analysis, cluster analysis, vertex component analysis) were tested with and without baseline correction. The best results have been achieved with VCA, an unsupervised method to rapidly unmix hyperspectral data. Given a set of mixed spectral (multispectral or hyperspectral) vectors, the algorithm aims at estimating the number of reference substances, also called endmembers, their spectral signatures, and their abundance fractions. Unsupervised endmember extraction by VCA exploits the following facts: the endmembers are the vertices of a simplex and the affine transformation of a simplex is also a simplex. Furthermore, VCA assumes the presence of pure pixels in the data. The algorithm iteratively projects data onto a direction orthogonal to the subspace spanned by the endmembers already determined. The new endmember signature corresponds to the extreme of the projection (Nascimento and Dias, 2005). Applied on plant cell walls the extreme of the projection gives the purest found spectra (components) of the map and at the same time the distribution of the different endmembers (components) within the map is plotted in the images. VCA was tested with and without baseline correction and using different wavenumber regions. To choose the right number of endmembers values from 2 to 8 have been tested and evaluated based on the resulting endmember spectra and abundance map. With the given knowledge on plant cell wall composition and spectral signatures the final number of endmembers was chosen to represent areas and spectra, which represent distinct areas and differences.

The extracted endmember spectra were exported into OPUS 7.0 (Bruker, Germany) and for detailed analysis baseline corrected (rubberband) and min-max normalized on the aromatic stretching vibration around 1600 cm^-1^.

## RESULTS

### SPRUCE EARLYWOOD AND LATEWOOD: UNIVARIATE IMAGING AND VERTEX COMPONENT ANALYSIS

**FIGURE 1:**
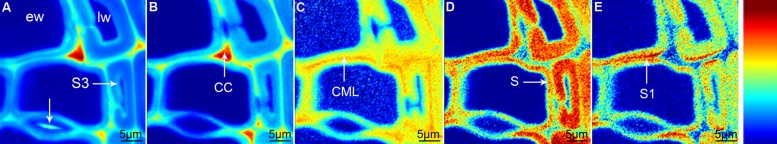
**Raman images of spruce wood cross sections (38 μm × 38 μm) based on univariate analysis: fluorescence visualized by plotting the intensity from 500 to 600 cm^-1^ (A), lignification by integration of the bands from 1711 to 1533 cm^-1^ (B), change in lignin composition by band height ratio of 1598/1657 cm^-1^ (C), cellulose content by band integration from 401 to 331 cm^-1^ (D), change in orientation of cellulose microfibrils by integration from 1106 to 1083 cm^-1^ (E)**.

Raman mapping gives a fluorescence image by plotting the intensity change of the background (**Figure 1A**), which in wooden cell walls in many cases looks similar like integration of the main lignin bands between 1711 and 1533 cm^-1^ (**Figure 1B**). Cell wall corner (CC) and compound middle lamella (CML) show higher intensity (red color) due to higher amount of lignin, which is the main cause of fluorescence. The main differences between the two pictures are that the pit membrane and the layer toward the lumen (S3) are emphasized in the fluorescence image (**Figure 1A**, arrows), but not in the lignin image (**Figure 1B**). An attempt to image not only changes in lignin amount, but also composition can be made by looking at the ratio of the intensity of the aromatic stretching vibration at 1599 cm^-1^ and the 1656 cm^-1^ band, assigned to C = C of coniferyl alcohol and C = O of coniferaldehydes (Agarwal et al., 2011; **Figure 1C**). By this the CC is no longer the region with highest intensity, but differs clearly together with CML from the secondary cell wall with a gradient toward the lumen (**Figure 1C**). Integration of the cellulose band at 380 cm^-1^ gives a clear picture of the secondary cell wall (**Figure 1D**) and due to changes in microfibril angle the S1 can be visualized by integrating the orientation sensitive band at 1095 cm^-1^ (Gierlinger and Schwanninger, 2006; **Figure 1E**). To really get a clear picture on chemistry and structure of the different visualized cell wall layers, underlying spectra have to be extracted for a detailed analysis (Gierlinger and Schwanninger, 2006; Richter et al., 2011). This can be time consuming and in many cases dependent on the personal view and therefore different multivariate approaches for image generation and spectra extraction have been explored.

**FIGURE 2:**
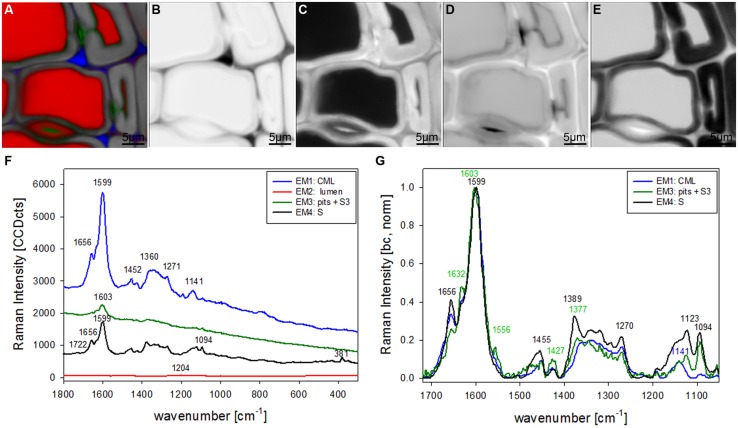
**Raman images of spruce wood cross sections (38 μm × 38 μm) based on VCA analysis with four endmembers using the wavenumber region from 1800 to 300 cm^-1^ :colored composite image visualizing all four endmembers (EM; A) and intensity changes of EM 1 corresponding to cell corners (B), EM 2 reflecting the lumen (C), EM 3 with high intensity in pit membrane and layer toward the lumen (S3; D), and EM 4 representing the secondary cell wall (E).** The corresponding endmember spectra are shown with original background and intensity **(F)** and after baseline correction and normalization on the aromatic stretching vibration at 1599 cm^-1^ **(G)**.

The most promising multivariate data analysis method on the investigated plant cell wall maps was the VCA. Choosing four endmembers (EM) on the before shown Raman map gives a clear separation in three different cell components [CML and CC, lumenside layer (S3) and pit membrane, secondary cell wall (S)] and the lumen (**Figure 2A**). Abundance maps for every EM show in detail the distribution of the different components (**Figures 2B–E**) with black color for high abundance and white color for no contribution. The corresponding four endmember spectra give information about composition and structure of the identified component and/or anatomical region (**Figures 2F,G**). The first EM spectrum (blue) shows a high fluorescence background and the main lignin bands at 1599, 1656 cm^-1^ and a couple of medium bands between 1500 to 900 cm^-1^ (**Figure 2F**). Intensity is highest and restricted to the cell corner and the middle lamella (**Figure 2B**). The second EM spectrum (red) represents the lumen (**Figure 2C**) and has the highest band at 1204 cm^-1^ from D_2_O and some smaller bands in the lignin region, which might correspond to residual very weak lignin contribution from the cell corner and middle lamella region. The third EM (green) reveals a different lignin spectrum, again with high background and small broad bands with the most intense at 1603 cm^-1^ (**Figure 2F**). This characteristic EM spectrum refers to the pit membranes and inner secondary cell wall (S3) layer (**Figure 2D**). The fourth EM spectrum (black) corresponds to the main secondary cell wall S2 (**Figure 2E**) and shows marker bands of lignin (1656 and 1599 cm^-1^), cellulose (1123, 1094, 381 cm^-1^), and hemicellulose (1722 cm^-1^). Comparison of baseline-corrected and on the aromatic stretching band around 1600 cm^-1^ normalized spectra shows the detailed changes due to changes in lignin composition and cellulose orientation (**Figure 2G**). The cellulose orientation sensitive doublet at 1123/1094 cm^-1^ shows a clear change between the S3 and pit (green, EM3) compared to the secondary cell wall (black, EM4; **Figure 2G**). The 1094 cm^-1^ band is much higher than the 1123 cm^-1^ band in the S3 and pit membrane (green, EM3). This means that the cellulose microfibrils are oriented with a high angle with respect to the fiber axis (Gierlinger et al., 2010b) in this region. The lignin composition changes between all three cell wall positions as can be seen by the different heights of the 1656 and 1632 cm^-1^ band as well as the change in peak position of the aromatic ring stretching vibration from 1599 in the thick S-layer to 1603 cm^-1^ in the S3 layer and pit membrane (**Figure 2G**). So from these VCA result we can conclude that the interface toward the lumen (S3) have similar lignin structure like the pit membrane and differ clearly from the S2 layer and CML. The 1655 cm^-1^ band, is reduced, whereas the band at 1632 cm^-1^, is enhanced. In the CML the two bands are in between the S3 and S2 layer. The 1452 cm^-1^ band, assigned to CH_3_ bending in OCH_3_ (Agarwal et al., 2011) is clearly increased in the S layer and lowest in the CML, but might be influenced by the increase of the underlying cellulose band at 1472 cm^-1^.

### BEECH FIBERS AND VESSELS: VERTEX COMPONENT ANALYSIS

**FIGURE 3:**
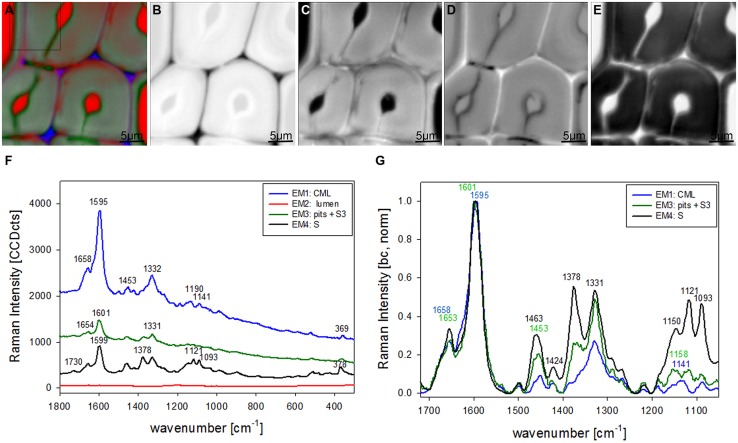
**Raman images of beech wood cross sections (30 μm × 30 μm) based on VCA analysis with four endmembers using the wavenumber region from 1800 to 300 cm^-1^ :colored composite image visualizing all four endmembers (EM; A) and intensity changes of EM 1 corresponding to cell corners (B), EM 2 reflecting the lumen (C), EM 3 with high intensity in pit membrane and layer toward the lumen (S3; D), and EM 4 representing the secondary cell wall (E).** The corresponding EM spectra are shown with original background and intensity **(F)** and after baseline correction and normalization on the aromatic stretching vibration at 1599 cm^-1^ **(G)**.

Vertex component analysis analysis of beech cross sections give on first hand similar Raman pictures separating the same three cell wall regions and the lumen (**Figures 3A–E**). Cell corners and CML are separated (**Figures 3A,B**) by the first EM and the spectrum shows again high fluorescence and mainly lignin bands (blue, **Figure 3F**). Due to the different lignin composition of hardwoods changes in the beech spectra are observed, e.g., the more pronounced band at 1332 cm^-1^ (**Figure 3F**), assigned to aliphatic O–H bending (Agarwal et al., 2011). Again the second EM represents mainly the lumen with D_2_O (**Figure 3C**) and the third EM reflects the pit membranes and linked connections to the inner layer (S3; **Figure 3D**). Compared to EM three in spruce (**Figure 2D**) more abundance within the thick secondary cell wall (medium gray) is observed and partly strong signal in the vessels (left upper and right lower corner, **Figure 3D**). The corresponding EM spectrum shows like in spruce quite high fluorescence (**Figure 3F**) and strong contributions from lignin (**Figures 3F,G**). The last EM describes the main secondary cell wall, having beside the lignin bands also clear signals from cellulose at 1378, 1121, 1093, 378 cm^-1^ and hemicellulose at 1730 cm^-1^ (**Figures 3F,G**). A zoom into the strong lignin region shows that in beech EM1, the CC and CML showed the highest shoulder around 1632 cm^-1^. Like in spruce the band at 1658 cm^-1^ is increased in the secondary cell wall (**Figure 3G**).

**FIGURE 4:**
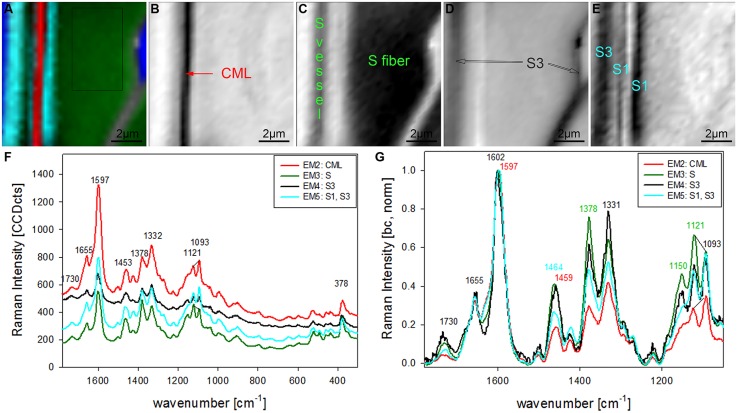
**Raman images of zoom into beech wood fiber and vessel wall (10 μm × 10 μm) based on VCA analysis with five endmembers using the wavenumber region from 1800 to 300 cm^-1^ :colored composite image visualizing all five endmembers (EM; A) and intensity changes of EM 2 corresponding to cell corners (B), EM 3 reflecting the secondary cell wall (C), EM 4 with high intensity in the layers toward the lumen (S3; D), and EM 5 representing the S1 and S3 layer (E).** The corresponding EM spectra are shown with original background and intensity **(F)** and after baseline correction and normalization on the aromatic stretching vibration at 1599 cm^-1^ **(G)**.

The chosen number of EMs in the VCA has an influence on the results as well as the selected area, wavenumber region or spectral pretreatment and has to be adjusted according to the research question. In the given examples four EMs have been shown to separate cell wall structures most different in lignin content and/or composition, when the analysis is based on untreated spectra from 300 to 1800 cm^-1^ (**Figures 2** and **3**). To reveal additional differences and heterogeneity of the cell wall of vessels and fibers a smaller region excluding the highly lignified cell corners and pit membranes was chosen (inset in **Figure 3A**) and analyzed with five EMs (**Figures 4A–G**). By this not only the lumen oriented S3 of the secondary cell wall was separated, but also the S1 within the fiber and the vessel wall (**Figure 4A**). All five EM spectra showed a similar intensity range and background as the highly lignified CC and pit region has been excluded (**Figure 4F**). The CML is clearly separated by the second EM (**Figure 4B**, first EM corresponds to the lumen and is not shown), which shows the highest lignification, but also contribution from cellulose bands (red, **Figure 4F**). The 1093 cm^-1^ band is much higher than the 1121 cm^-1^ band pointing to alignment of the cellulose fibrils with a high angle in respect to the fiber axis (Gierlinger et al., 2010a). The third EM describes the main secondary cell wall of the fiber and partly the vessel (**Figure 4C**) with the lowest lignin content and cellulose microfibrils aligned parallel to the fiber axis, as expressed by the ratio of the 1121 and 1093 cm^-1^ band (green, **Figures 4F,G**). The fourth EM is again the lumen-sided S3 layer (**Figure 4D**) with high fluorescence (black, **Figure 4F**) and more noisy slightly changed lignin bands (**Figure 4G**). The fifth EM now clearly separates the S1 layer of the fiber and vessel, but also the lumen part of the vessel wall (S3, **Figure 4E**). These regions are described by slightly higher lignin content than the main secondary cell wall and a high microfibril angle (with respect to the fiber axis, **Figures 4F,G**). So it can be concluded that most of the vessel wall is ascribed to higher lignin content and higher microfibril angle with respect to the fiber axis (cyan EM5 and black EM4, **Figures 4D,E**) than the secondary cell wall of the fiber (red EM2, **Figure 4C**).

**FIGURE 5:**
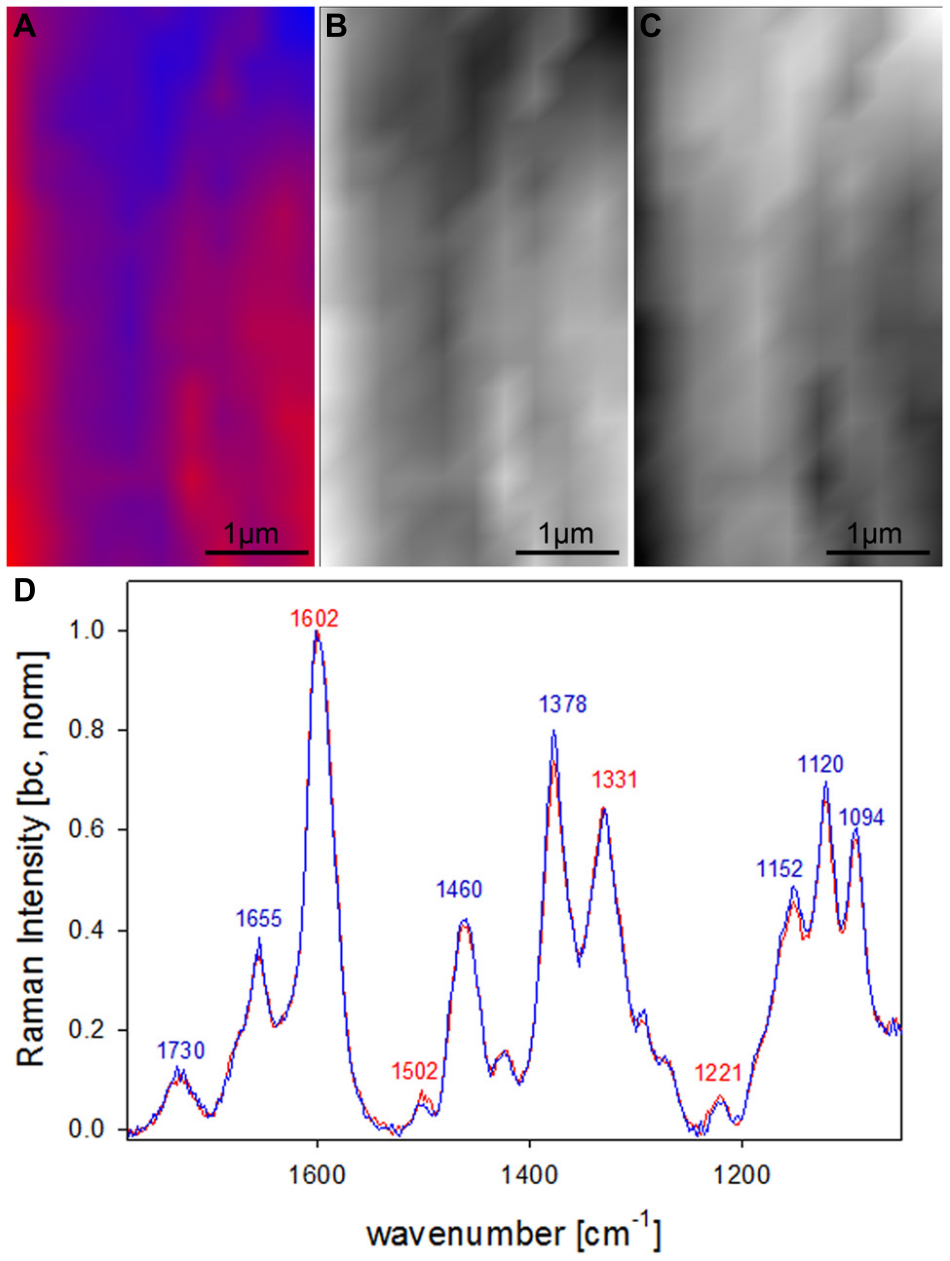
**Raman images of zoom into beech wood fiber secondary cell wall (4 μm × 7 μm) based on VCA analysis with two endmembers using the wavenumber region from 1800 to 300 cm^-1^ :colored composite image visualizing a lamellar structure (A) and intensity changes of endmember 1 (EM1; B) and EM2 (C) and the corresponding EM spectra after baseline correction and normalization on the aromatic stretching vibration at 1599 cm^-1^ (D)**.

After clear distinction of the cell wall layers (CC-CML-S1-S2-S3) based on changes in molecular structure, the question arised if the S2 fiber cell wall is really of uniform molecular composition or if VCA could visualize also subtle differences by analyzing solely the S2 layer. Therefore in a next step a zoom into the secondary cell wall (inset in **Figure 4A**) was analyzed using two EMs (**Figures 5A–D**). By this a lamellar structure was revealed (**Figures 5A–C**) with slightly changing lignin amount and composition (**Figure 5D**). Although the spectral (molecular) changes are small these lamellar structure have been revealed at different positions (zooms) and independent Raman maps.

## DISCUSSION

### UNIVARIATE VERSUS MULTIVARIATE DATA ANALYSIS METHODS

The Raman imaging approach based on univariate data analysis (**Figure 1**) gives quite important insights into plant cell wall design, but has some drawbacks. To relate intensity changes of a certain band directly to changes in the amount of a specific single cell wall component or functional group is only possible if intensity changes due to different focal points (changes in sample height) can be excluded and the band of interest has no overlap with other bands attributed to different components. By preparing very plane sample surfaces or by plotting ratios of selected bands the intensity changes due to different sample heights (focus) can be eliminated. Nevertheless the following problems of univariate analysis remain: (1) plant cell wall spectra are characterized by broad overlapping bands and only a few marker bands which are not influenced by neighboring and/or underlying bands and/or shoulders of other components or functional groups are present (2) not only the amount, but also changes in polymer composition and/or form (e.g., crystallinity) and/or orientation are influencing Raman band shape and intensity. Therefore interpretation of images based on univariate data analysis should always include detailed analysis and/or control of the underlying spectra. Yet this is often neglected, because average or characteristic spectra of selected regions are not automatically calculated by the software. Furthermore calculating average spectra can be time consuming and region selection or intensity threshold definition depends on the personal view and the beforehand calculated image.

Multivariate methods overcome the problem of overlapping bands as the whole spectra or wider spectral regions are analyzed. Additionally to the derived Raman images the corresponding average spectra (cluster analysis), loadings (Principal component analysis) or endmembers (VCA) are available for data interpretation. The three mentioned multivariate approaches have been tested on different plant cell wall Raman maps and as VCA gave the best results only these have been shown here. Best results are considered as coming up with the most reasonable distinction of regions and/or layers and spectra or loadings or endmembers, which represent most pure component or layer spectra. Another advantage has been the fact that good results have been achieved even without baseline correction, which was not the case for most of the other approaches.

In the univariate analysis CC and CML could be visualized due to the changes in the lignin band (**Figures 1B,C**) and the S1 and the whole S wall by integrating cellulose bands (**Figures 1D,E**). The S3 and pit-membrane were only visible in the fluorescence image (plotting intensity change in a region without a band, **Figure 1A**). The VCA analysis revealed for the first time that pit membranes and the inner S3 layer have a similar molecular structure, which differs clearly from all other regions (**Figures 2D** and **3D**). Furthermore a lamellar structure within the secondary cell wall has been visualized by Raman VCA imaging (**Figure 5**). VCA clearly finds the most relevant and also subtle molecular changes within one image scan (4.2) and additionally opens up new opportunities for comparing different samples based on the EM spectra (4.3).

### CHANGES IN LIGNIN AMOUNT AND COMPOSITION IN DIFFERENT CELL WALL REGIONS

Vertex component analysis images and EM spectra clearly demonstrated that not only the amount of lignin is changing between the different cell wall regions and layers, but also lignin composition. It is already known that the amount and chemical characteristics of lignin vary across the cell wall, with the presence of reaction wood and among cell types (Donaldson, 2001). It is difficult to perform reliable chemical analysis of lignin from separate layers in wood as many different cell types and tissues are present and fractionations tend to include mixtures and thus give uncertain results. Therefore a variety of microscopy techniques including microautoradiography, histochemistry, UV absorbance, interference microscopy, fluorescence and transmission electron microscopy have been applied. The higher amount of lignin in CML and CC compared to the secondary cell wall was verified using many different methods (Donaldson, 2001). By selective labeling of *p*-hydroxyphenyl-guaiacyl and syringylpropane moieties the growing process of protolignin macromolecule in specific morphological region was visualized by high resolution microautoradiography and the content of condensed lignin is reported to be higher in the cell corner and middle lamella than in secondary cell wall lignin (Terashima and Fukushima, 1988). The normalized EM spectra corresponding to the two different anatomical regions (**Figures 2G** and **3G**) confirm this result by a relative decrease of the band at 1656 cm^-1^ (spruce) and 1658 cm^-1^ (beech), which is assigned to C = C of coniferyl alcohol and C = O of coniferaldehydes (Agarwal et al., 2011) in the CML compared to the secondary cell wall. In spruce the EM corresponding mainly to the pits (and partly S3 layer; green line **Figure 2G**) shows an even higher decrease of the 1656 cm^-1^ band which might be explained by the fact that lignification starts in the middle lamella between pit borders (Kutscha and Schwarzmann, 1975) and thus probably representing the oldest developmental stage of lignification within the scanned area. As the maps are within the last two annual growth rings of fresh never dried-out sapwood the question arises if lignin polymerization is already completely finished or not. The method thus will have the potential to clarify the lignin polymerization process by a detailed study from cambium to the sapwood/heartwood border.

Together with the described band decrease a band arises at 1632 cm^-1^ in the pit (S3) EM spectra (green line **Figure 2G**). An increase of a band at 1630 cm^-1^ was reported in anti-sense CAD clones of tobacco plants and attributed to incorporation of coniferaldehyde like moieties into the lignin structure (Stewart et al., 1997). Coniferaldehyde can be the direct precursor of coniferyl alcohol, but is also known to be formed in the course of dehydropolymerization of coniferyl alcohol (Boerjan et al., 2003; Higuchi, 2006). Possibly also stilbenes contribute in this region, as a marker band is reported at 1635 cm^-1^ (Agarwal and Atalla, 2000). The broadening and shift to higher wavenumber of the aromatic stretching vibration in the pit and S3 membran may also suggest the formation of *o*- and *p*-quinone type structures, like reported in the phodegradation study of wood (Pandey and Vuorinen, 2008). Also the shoulder/band at 1556 cm^-1^ could be attributed to *o*-quinone (Agarwal and Atalla, 2000). The high fluorescence in relation to the low band intensity (**Figures 2F** and **3F**, green line) probably comes from chromophores and a more conjugated system over the aromatic rings (Lahdetie et al., 2013). So we can conclude that more oxidized lignin moieties are found in the surface layers represented by pit membrane and S3 layer. The degree of oxidization is suggested to have an effect on function and treatment of woody structures and is thus of great research interest. So far the S3 layer has often been reported to be more highly lignified than the adjacent S2 layer (Donaldson, 1987), but depending on the technique results on increased content might also be influenced by changes in lignin composition.

In spruce the earlywood has higher contribution in EM3 (**Figure 2D**) than the latewood and in beech the vessel higher than the fiber (**Figure 3D**). As the changed lignin composition in pits and S3 is more pronounced in the tissue (earlywood) and elements (vessels) specialized on water conduction in the tree, a role or support for this functionality is suggested. The band characteristic for syringyl units at 1331 cm^-1^ clearly increased in the secondary cell wall EM spectra (**Figure 3G**, green and black line) compared to the CML (**Figure 3G**, blue line). This is in accordance with results that syringyl lignin are deposited especially during the late stages of secondary cell wall formation in hardwood fibers (Ruel et al., 1999).

#### Lamellar structure in beech

By restricting the VCA analysis on a small region within the S2 layer of the thick walled fibers a lamellar structure was revealed (**Figures 5A–C**). The endmember spectra showed that these differentiation is based on subtle changes of the lignin content and composition (**Figure 5D**). Although these spectra are far away from pure component spectra and changes are small, a lamellar structure was revealed, which has not been seen by univariate approaches. Similar segmented circumferential nanostructure in the 100 nm range have been seen recently by applying for the first time near-field scanning optical microscopy (SNOM) on secondary plant cell walls. Based on comparative analysis of model substances the elucidated nanoscale structure was suggested to reflect variations in lignification within the secondary cell wall (Keplinger et al., 2014). Within this study VCA Raman imaging proved the assumption of lamellar changes of lignin composition and content, which probably is due to non-uniform lignification during growth.

### COMPARING DIFFERENT SAMPLES BASED ON ENDMEMBER SPECTRA

Based on the EM spectra it is now possible to compare samples (in our case beech and spruce) based on the found extreme projections (EM spectra). If comparison is the aim of the study care should be taken to use parameters (wavelength region, selected sample area, number of endmembers) that reveal the same distinguished cell wall areas (e.g., in the shown example: CML, S, pits, and S3). This enables a comparison based on the selected regions, which will bring new insights into the effect of different modifications (e.g., genetic or chemical). Not only the general change can be probed, but information whether the changes are uniform or selected parts of the cell wall are more or less affected.

The first EM spectra of spruce and beech have been identified as almost pure lignin spectra as no contributions from carbohydrates are seen (**Figures 2F,G** and **3F,G**). So VCA enabled to extract spectra from native softwood and hardwood lignin by looking at the most extreme projections in the dataset, which are located as expected in the CC (**Figures 2B** and **3B**). Comparing the two shows clearly that intensity and also the background was higher in spruce (**Figures 2F** and **3F**), which is explained by the different lignin amount and structure. When comparing the normalized lignin spectra clear differences are seen in band intensities and positions (**Figure 5A**). The difference in intensity comes from higher lignin amount in spruce and different concentration of aromatic ring-conjugated structures. This is supported by a decrease of the 1630 cm^-1^ shoulder assigned to coniferylaldehyde/sinapaldehyde and the 1658 cm^-1^ band, where also coniferyl alcohol/sinapyl alcohol contribute. The higher cinnamaldehyd structure content in spruce is also supported by the higher band at 1140 cm^-1^. The aromatic ring stretching vibration changes position from 1595 cm^-1^ in beech to 1599 cm^-1^ in spruce in accordance with Raman spectra of milled-wood lignins of softwood and hardwood (Agarwal et al., 2011).

**FIGURE 6:**
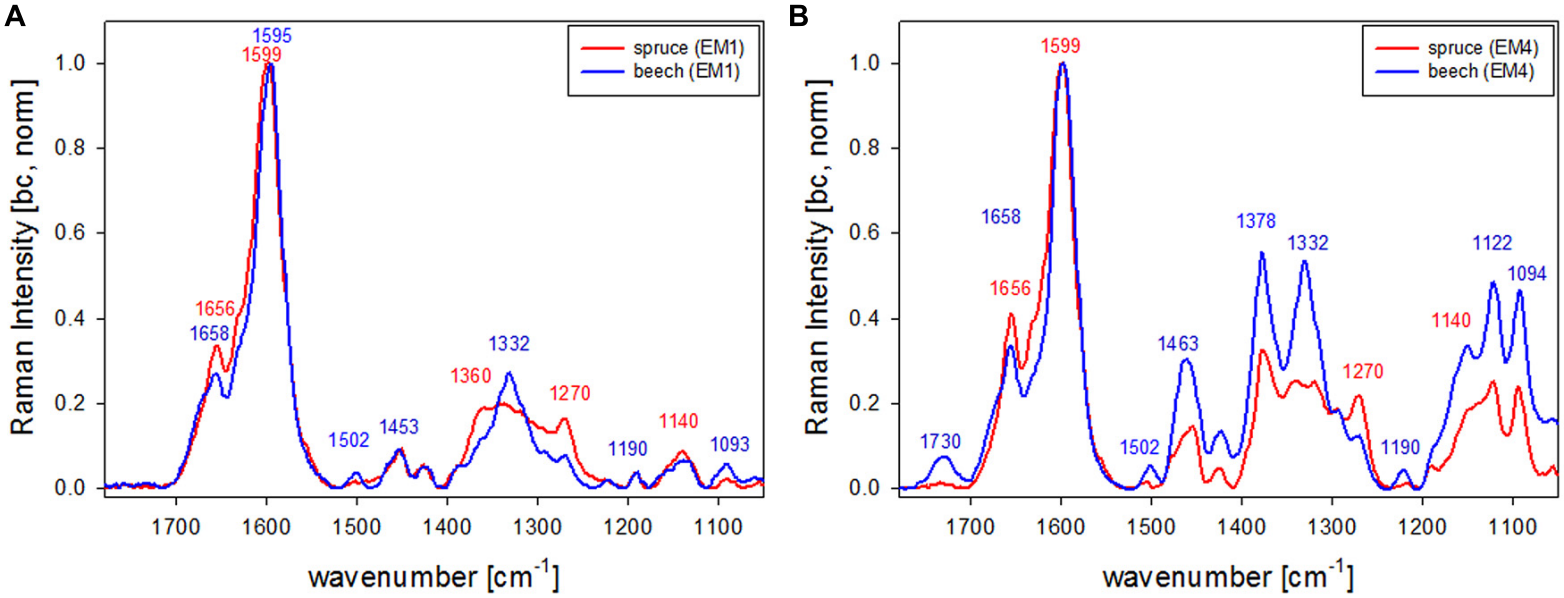
**Comparison of Raman spectra of spruce and beech after baseline correction and normalization on the aromatic stretching vibration at 1599 cm^-1^ based the analysis shown in **Figures 2** and **3** representing CC and CML (EM1, A) and the secondary cell wall (EM4, B; red line: spruce, blue line: beech)**.

Besides typical higher bands in spruce are at 1360 cm^-1^ (CH-bending in R3C-H), 1294 cm^-1^ (Aryl-*O* of aryl-OH and aryl-*O*-CH_3_, C–C stretch of coniferyl alcohol) and 1270 cm^-1^ [Aryl-*O* of aryl-OH and aryl-*O*-CH_3_, guaiacyl/syringyl ring (with C = O group) mode], while the 1332 cm^-1^ (aliphatic O–H bending) band is more characteristic and sharp in beech. The 1332 cm^-1^ band is therefore often used as a marker band for syringyl units, whereas the 1270 band is taken for guaicyl (Sun et al., 2011b). The overlapping nature in this band region is seen in the pure lignin spectra, especially in softwoods (**Figure 6A**). Looking at the spectra of secondary cell wall (**Figure 6B**, EM4) it becomes clear that not only the lignin modes overlap, but additionally the cellulose bands at 1378 and 1342 cm^-1^ contribute in this region and make a straightforward accurate determination of S/G ratio using these two bands and spectral fitting quite challenging, especially when cellulose content changes too. Nevertheless by including samples with quite different chemistry (*Eucalyptus*, maize, *Sorghum*, *Arabidopsis*, switchgrass) and ratios (0.04–1.68) a calibration curve was established by Sun et al. (2011b).

What is also clearly seen in the comparison of the secondary cell wall of spruce and beech (**Figure 6B**) is the change in hemicellulose composition. While in spruce almost no signal is found at 1730 cm^-1^, a clear band evolved in the beech, which is attributed to ester carbonyl groups from xylans (Lewis et al., 1994).

Investigating the Raman spectrum over the whole spectral range with VCA gives clearly the advantage that all polymers are studied at once and in terms of all aspects: orientation, content, and composition. VCA imaging has proven to be able to visualize also subtle differences in molecular structure. The extracted endmember spectra included only one pure component spectrum of lignin from the cell corner regions. All others represent mixtures, which are characteristic for the different resolved spatial areas and thus give detailed information on the changes in the molecular structure between these regions and different species.

## Conflict of Interest Statement

The author declares that the research was conducted in the absence of any commercial or financial relationships that could be construed as a potential conflict of interest.
